# Lacidipine Ameliorates the Endothelial Senescence and Inflammatory Injury Through CXCR7/P38/C/EBP-β Signaling Pathway

**DOI:** 10.3389/fcvm.2021.692540

**Published:** 2021-07-06

**Authors:** Xing Liu, Zhuoshan Huang, Yuanyuan Zhang, Xing Shui, Fanmao Liu, Zhen Wu, Shiyue Xu

**Affiliations:** ^1^Department of Hypertension and Vascular Disease, The First Affiliated Hospital, Sun Yat-sen University, Guangzhou, China; ^2^Department of Cardiology, The Third Affiliated Hospital, Sun Yat-sen University, Guangzhou, China; ^3^National-Guangdong Joint Engineering Laboratory for Diagnosis and Treatment of Vascular Diseases, Guangzhou, China; ^4^National Health Commission Key Laboratory of Assisted Circulation, Sun Yat-sen University, Guangzhou, China

**Keywords:** lacidipine, oxidative stress, senescence, inflammation, CXCR7

## Abstract

**Background:** Lacidipine, a third-generation calcium channel blocker, exerts beneficial effects on the endothelium of hypertensive patients in addition to blood pressure lowering. However, the detailed mechanism underlying Lacidipine-related endothelial protection is still elusive.

**Methods:** Sixteen spontaneous hypertensive rats (SHRs) were randomly divided into two groups: Lacidipine-treated SHR group and saline-treated control group. Tail systolic blood pressure was monitored for four consecutive weeks. Endothelial cells (ECs) were pretreated with Lacidipine prior to being stimulated with H_2_O_2_, bleomycin, or Lipopolysaccharides (LPS) *in vitro*. Then, cell activity, migration, and senescence were measured by Cell Counting Kit-8 assay, transwell assay, and β-galactosidase staining, respectively. The fluorescent probe 2′, 7′-dichlorofluorescein diacetate (DCFH-DA) was used to assess the intracellular reactive oxygen species (ROS). Related protein expression was detected by Western blotting and immunofluorescence.

**Results:** Our data showed that Lacidipine treatment lowered the blood pressure of SHRs accompanied by the elevation of CXCR7 expression and suppression of P38 and CCAAT/enhancer-binding protein beta (C/EBP-β) compared with the control group. *In vitro* experiments further demonstrated that Lacidipine increased the cell viability and function of ECs under oxidative stress, cell senescence, and inflammatory activation *via* the CXCR7/P38/signaling pathway.

**Conclusions:** Our results suggested that Lacidipine plays a protective role in EC senescence, oxidative stress, and inflammatory injury through the regulation of CXCR7/P38/C/EBP-β signaling pathway.

## Introduction

Hypertension is recognized as a leading risk factor for cardiovascular diseases due to its effects on vascular injury (1). Moreover, endothelial dysfunction is considered to be the initial step of hypertension-related vascular injury (2, 3). Emerging evidence suggests that oxidative stress, inflammation activation, and cell senescence play crucial roles in the pathogenesis of hypertension-related endothelial dysfunction (4–7). Hence, it is important to decipher the detailed mechanisms underlying hypertension-related endothelial cell (EC) injury and dysfunction in order to improve the prognosis of hypertensive patients.

Studies showed that CXC chemokine receptor 7 (CXCR7) plays a key role in the modulation of cellular oxidative damage and senescence (8). Suppression of CXCR7 partially reduces the *in vitro* functions and *in vivo* re-endothelialization capacity of endothelial progenitor cells (EPCs) from hypertensive patients (9). Moreover, the CXCR7/p38 axis has been reported to be highly involved in the protection against high glucose-induced EPC dysfunction (10). In addition to oxidative stress, inflammatory injury usually occurs in the pathogenesis of hypertension (11) of which CCAAT/enhancer-binding protein beta (C/EBP-β) is a critical regulator (12). However, whether CXCR7/P38/C/EBP-β participated in the regulation of endothelial senescence and inflammatory injury remains to be uncovered. Lacidipine, a third-generation calcium channel blocker, has been demonstrated to be effective for preventing endothelial dysfunction in salt-loaded stroke-prone hypertensive rats (13) and restoring endothelial-dependent vasodilation in hypertensive patients (14). In addition to calcium channel-modulated vasodilation, Lacidipine displays an antioxidant activity greater than other dihydropyridine calcium antagonists (15) and exerts an anti-inflammatory effect in carrageenan model in intact rats (16). Our previous study demonstrated that Lacidipine improved the endothelial repair capacity of EPCs from patients with essential hypertension (17). Thus, we hypothesized that Lacidipine may attenuate endothelial senescence and inflammatory injury by modulating the CXCR7/P38/C/EBP-β pathway.

To validate our hypothesis, the *in vivo* effects of Lacidipine on CXCR7/P38/C/EBP-β were assessed by using spontaneous hypertensive rats (SHRs). Then, the detailed mechanisms by which Lacidipine affected oxidative damage, cell senescence, and inflammatory activation *via* the CXCR7/P38/C/EBP-β pathway in ECs were further investigated *in vitro*.

## Materials and Methods

### Experimental Animals

A total of 16 SHRs were obtained from the Vital River Laboratory Animal Technology Co., Ltd. (Beijing, China) and housed in room temperature (~25°C) on 12 h day/12 h night cycles, which had free access to standard chow diet and water. For the experimental requirement, all SHRs were randomly assigned into two groups, including Lacidipine-treated group (*n* = 8, rats were intragastrically treated with Lacidipine at a dose of 3 mg/kg/d for 4 weeks) and control group (*n* = 8, rats were intragastrically treated with physiologic saline for 4 weeks). Systolic blood pressure (SBP) was measured with a non-invasive computerized tail-cuff method and obtained by averaging 10 measurements. Meanwhile, their body weights were measured each week. After 4 weeks, all rats were sacrificed under isoflurane (2%) anesthesia. Aortic tissues were acquired for further analysis. All animal experimental protocols complied with institutional guidelines and were reviewed and approved by the Animal Care and Use Committee of the Ethics Committee of the First Affiliated Hospital of Sun Yat-sen University (Guangzhou, China).

### EC Culture and Treatment

Human aortic endothelial cells (HAECs; Cambrex, Walkersville, MD, USA) from passage three to eight were cultured in Endothelial Growth Medium 2 (EGM-2; Lonza, Allendale, NJ, USA) with 20% fetal bovine serum (FBS; Hyclone, South Logan, UT, USA) in a 5% CO_2_ incubator at 37°C. To simulate oxidative damage and senescence, ECs were stimulated with H_2_O_2_ (100 μM for 1 h) and bleomycin (50 μg/ml for 3 h), respectively, following Lacidipine pretreatment (100 nM for 12 h). C/EBP-β expression vector pcDNA3.0-C/EBP-β and small interfering RNA-CXCR7 (si-CXCR7: 5′-ATCAAATGACCTTGGATACTG-3′) were synthesized by RiboBio Co., Ltd. (Guangzhou, China) and transfected into ECs through a lipofectamine 2000 kit (Invitrogen, Carlsbad, CA, USA).

### CCK-8 Assay

Cell viability was assessed by performing the Cell Counting Kit-8 (CCK-8) assay. In brief, the cells in each group were made into a cell suspension and seeded into 96-well plates at a density of 3,000 cells per well. Afterwards, the cells in each well were incubated with 10 μl of CCK-8 solution at 37°C for 2 h. The absorbance at a wavelength of 450 nm was measured using a microplate reader.

### β-Galactosidase Staining

ECs were harvested and washed after treatment. Then, the cells were fixed with 2% formaldehyde and subsequently stained with a Senescence β-Galactosidase Staining Kit (CST, Boston, MA, USA). Level of senescence was quantified by visual examination of dark blue stained cells with an inverted microscope (Nikon, Japan) at 200 × magnification.

### ROS-Level Measurement

Intracellular reactive oxygen species (ROS) was determined with the non-fluorescent probe 2′,7′-dichlorofluorescein diacetate (DCFH-DA; Beyotime Institute of Biotechnology, China) according to the manufacturer's instructions. Briefly, the cells at a density of 1 × 10^5^ cells per well were plated into six-well plates and incubated with 20 μM DCFH-DA for 30 min at 37°C. ROS generation was analyzed by flow cytometric analysis.

### Detection of NADPH Activity

The cells from different groups were harvested and applied into NADPH oxidase activity analysis following the instructions provided by Beyotime Institute of Biotechnology (Shanghai, China). The NADPH activity was expressed as μmol/min/mg.

### EC Migration *in vitro*

Transwell assay was performed to analyze cell migration. In brief, approximately 200 μl of serum-free medium containing 2 × 10^4^ ECs was seeded into the upper chamber (8 μm pore size; Corning, NY, USA), whereas 500 μl of medium containing 10% serum was added to the lower chamber. After 24 h of incubation at 37°C, the cells that migrated into the lower chamber were fixed with 4% paraformaldehyde for 15 min, stained with 1% crystal violet (#C0775; Sigma-Aldrich, Milwaukee, WI, USA) for 30 min, and counted by independent investigators.

### Immunofluorescence

Briefly, the cells were cultured on coverslips, washed with cold phosphate-buffered saline (PBS), and fixed for 30 min with 95% ethanol. After permeabilization in 0.5% Triton X-100, the cells were incubated with primary antibodies against CXCR7 (1:500; Abcam, Cambridge, MA, USA), followed by incubation with secondary antibody Alexa Fluor 488 goat anti-rabbit (Cwbiotech, Beijing, China). Next, the cells were washed three times in the dark for 1 h and counterstained with DAPI (Solarbio, Beijing, China). The fluorescent signal was analyzed under a fluorescence microscope (Nikon, Japan).

### Apoptosis Analysis

The cells from different groups were harvested, seeded onto six-well plates, and cultured overnight. On the next day, the cells were incubated with Annexin V-FITC/PI Apoptosis Kit (#KA3805; Abnova, Shanghai, China) according to the manufacturer's instructions. Apoptotic cells were examined using flow cytometry (BD Biosciences, San Jose, CA, USA) and confirmed to be a positive Annexin V-FITC signal.

### Western Blot

RIPA lysis buffer (Beyotime Institute of Biotechnology, Haimen, China) was used to extract all protein samples from tissues or cells according to the manufacturer's instructions. After quantitation by BCA assay, an equal amount of protein sample was separated on SDS-PAGE and then transferred onto PVDF membranes. Afterwards, the membranes were incubated with primary antibodies against CXCR7, NOX1, NOX2, C/EBP-β, P38, p-P38, VCAM1, Endoglin, MCP-1, IL-6, NLRP3, caspase-1 (all Abcam), and GAPDH overnight at 4°C. Then, the membranes were incubated with HRP-conjugated secondary antibodies for 2 h at room temperature, and protein bands were detected with ECL reagents (#131023-60-4; BOC Sciences, Shirley, NY, USA) with GAPDH as the internal control.

### Statistical Analysis

All quantitative data were expressed as mean ± standard deviation (SD) of at least three independent experiments. Statistical analysis was performed with GraphPad Prism software 6.0. Student's *t*-test was used to analyze differences between two groups, whereas one-way analysis of variance followed by Tukey's *post-hoc* test was utilized to compare differences among multiple groups. All values with *p* < 0.05 indicated a significant difference.

## Results

### Lacidipine Attenuates Hypertension and Regulates CXCR7/P38/C/EBP-β Expression *in vivo*

**Figure 1:**
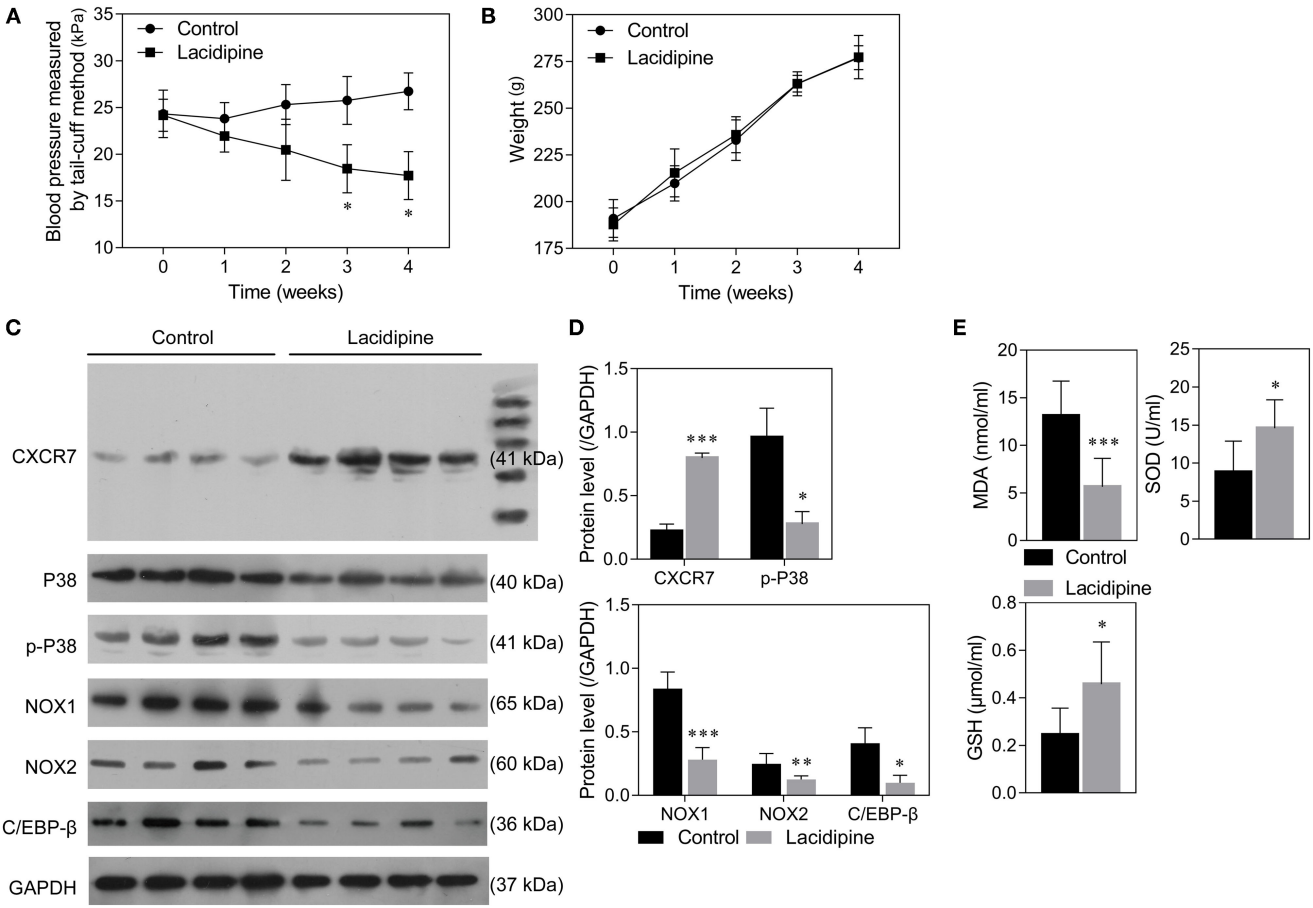
Lacidipine attenuated arterial hypertension and regulated CXCR7/P38/C/EBP-β expression *in vivo*. Blood pressure of spontaneously hypertensive rats (SHRs) treated with and without Lacidipine was measured by the tail-cuff method **(A)**. (Data are mean ± SD, *n* = 8 in each group). The weight of SHRs in the control and Lacidipine groups **(B)**. Western blot was performed to analyze the protein expression of CXCR7, P38, p-P38, C/EBP-β, NOX1, and NOX2 in the aortic tissues from the control and Lacidipine groups **(C,D)**. The levels of MDA, SOD, and GSH in serum were measured by ELISA assays **(E)**. (^*^p < 0.05, ^**^p < 0.01, ^***^p < 0.001, compared with the control group).

Our previous study showed that Lacidipine treatment improved the endothelial repair capacity of EPCs from hypertensive patients. To further explore the molecular mechanism underlying the protective role of Lacidipine against hypertension, SHRs were treated with or without Lacidipine for 4 weeks. Our data showed that Lacidipine treatment significantly reduced the blood pressure in SHRs (Figure 1A) but did not significantly affect the weight of SHRs (Figure 1B). The expression of CXCR7 was significantly elevated in the aortic tissues isolated from SHRs after Lacidipine treatment. On the contrary, P38 expression and its phosphorylation, as well as the protein expression of C/EBP-β and NADPH oxidases (NOX1 and NOX2), were suppressed in the Lacidipine group compared with the control group (Figures 1C,D). Furthermore, Lacidipine reduced the level of malondialdehyde (MDA) in serum, whereas it significantly increased the levels of superoxide dismutase (SOD) and glutathione (GSH) (Figure 1E).

### Lacidipine Enhances the *in vitro* Function of ECs Under Oxidative Stress

**Figure 2:**
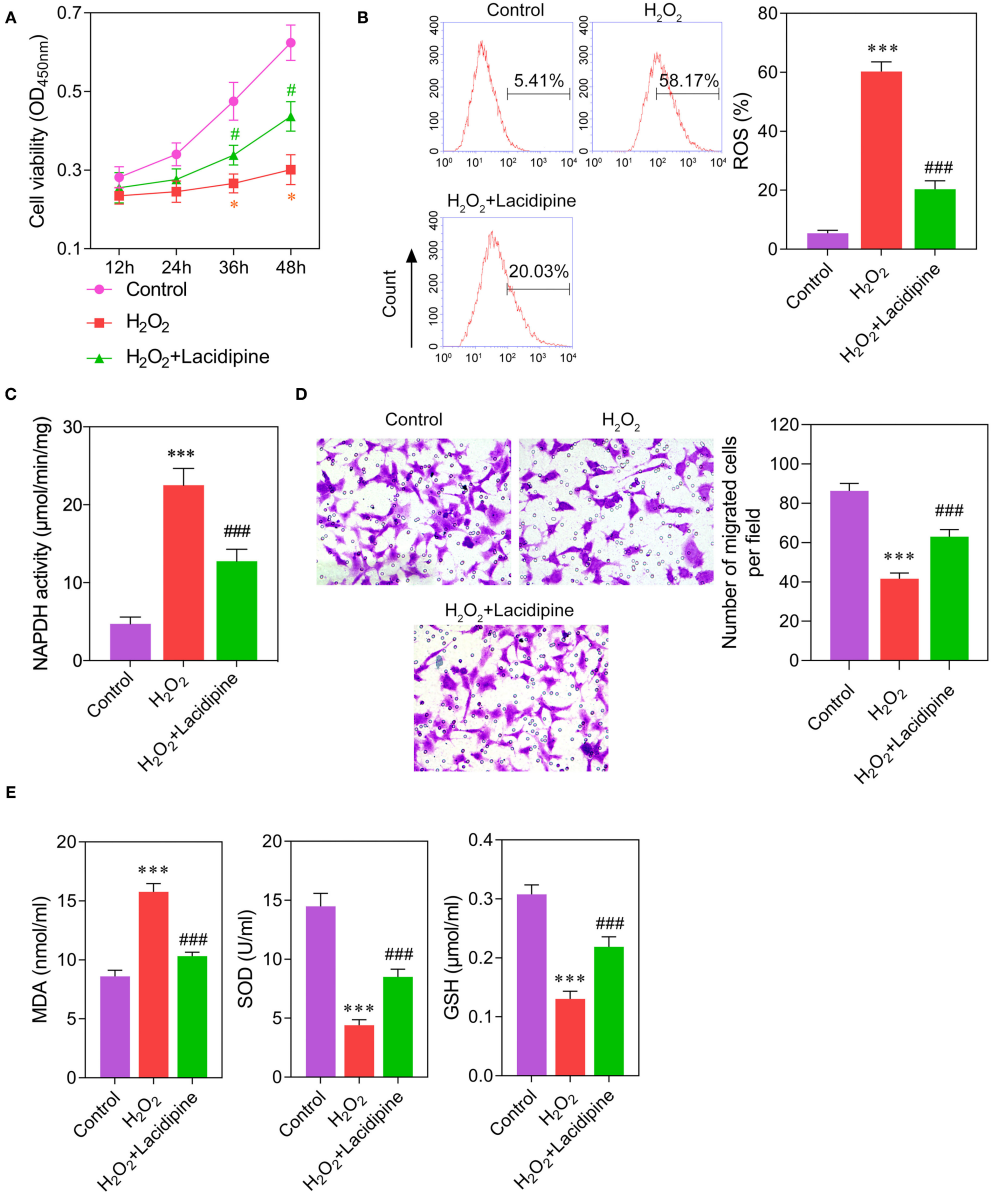
Effects of Lacidipine on the *in vitro* function of ECs under oxidative stress. Cultured ECs were treated with Lacidipine, followed by H_2_O_2_ stimulation (ROS induction). Cell viability was determined using CCK-8 assay **(A)**. ROS generation was assessed by flow cytometry with 2′,7′-dichlorofluorescein diacetate **(B)**. NADPH activity was measured in different groups **(C)**. Transwell assay was applied to analyze cell migration ability **(D)**. The levels of MDA, SOD, and GSH in culture supernatant were detected by ELISA assays **(E)**. (Data are mean ± SD of three independent experiments. *p < 0.05, ***p < 0.001, compared with the control group; #*p* < 0.05, ###*p* < 0.001, compared with the H_2_O_2_ group).

To further explore the effect of Lacidipine on the oxidative damage in ECs, cultured ECs were treated with Lacidipine, followed by H_2_O_2_ stimulation (ROS induction). Our results showed that H_2_O_2_ stimulation significantly impaired cell viability (Figure 2A) and migration (Figure 2D) *in vitro* that were notably ameliorated by Lacidipine treatment. Moreover, Lacidipine treatment significantly suppressed the mitochondrial ROS production (Figure 2B), NADPH activity (Figure 2C), and MDA level induced by H_2_O_2_ in ECs. In contrast, the levels of SOD and GSH were significantly higher in the H_2_O_2_ + Lacidipine group than in the H_2_O_2_ group (Figure 2E). These results suggested that Lacidipine treatment attenuated the oxidative damage induced by H_2_O_2_ in ECs.

### Lacidipine Ameliorates the Senescence of ECs Induced by Bleomycin

**Figure 3:**
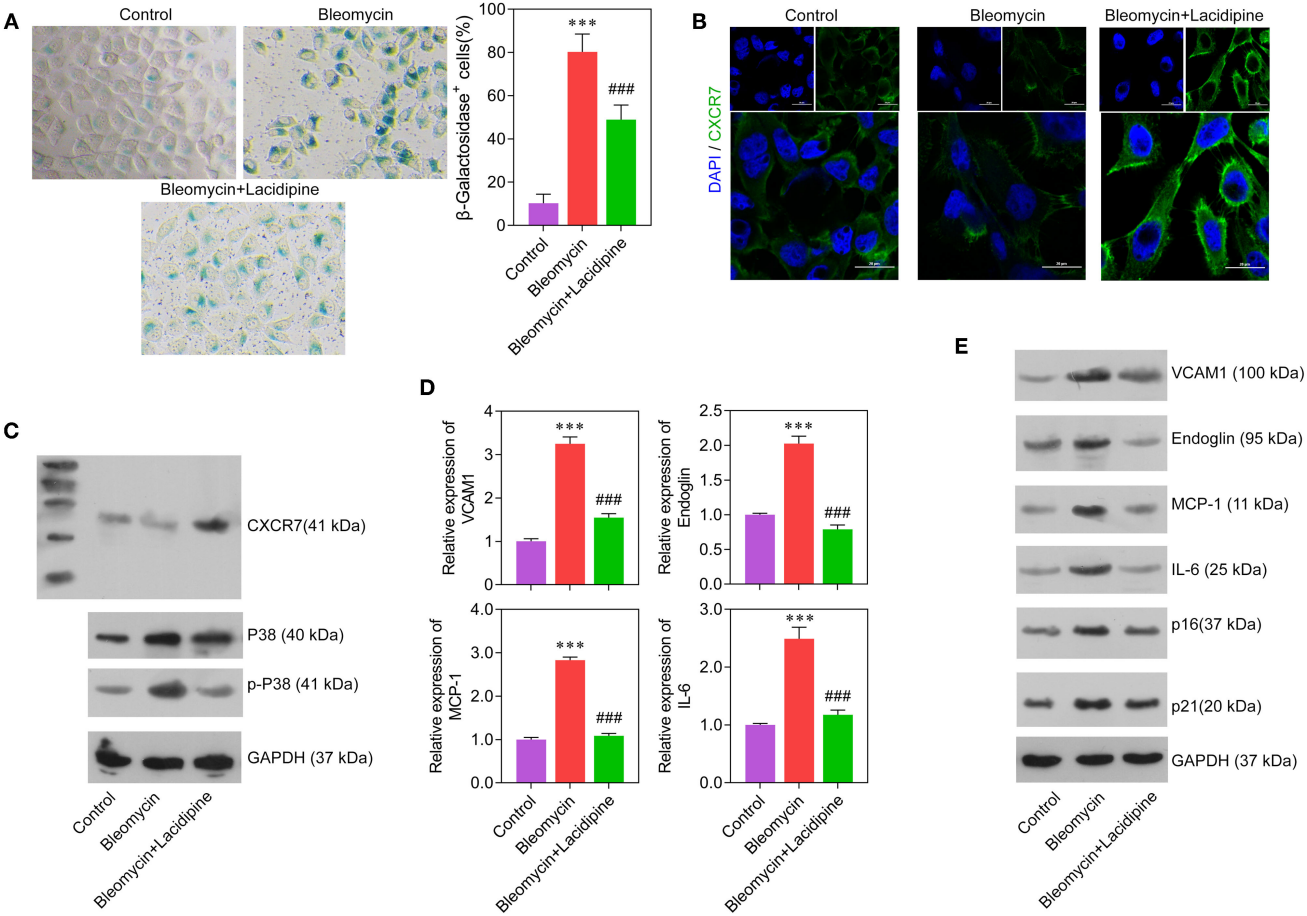
Lacidipine inhibited endothelial senescence and promoted CXCR7 expression. Cultured ECs were treated with Lacidipine, followed by bleomycin stimulation (senescence induction). Cell senescence was determined through β-galactosidase staining **(A)**. Immunofluorescence assay was applied to determine the protein expression of CXCR7 **(B)**. Western blot was performed to analyze the protein expression of CXCR7, P38, and p-P38 **(C)**. qPCR **(D)** and Western blot were performed to analyze the levels of VCAM1, Endoglin, MCP-1, IL-6, P16, and P21 **(E)**. (Data are mean ± SD of three independent experiments. ***p < 0.001, compared with the control group; ###*p* < 0.001, compared with the bleomycin group).

To determine the role of Lacidipine in the modulation of EC senescence, cultured ECs were treated with Lacidipine, followed by bleomycin stimulation. β-Galactosidase staining showed that Lacidipine treatment partially attenuated the senescence of ECs induced by bleomycin stimulation (Figure 3A). This result was further confirmed by Western blot analysis of cell senescence markers P16 and P21 (Figure 3E). Furthermore, the protein level of CXCR7 was decreased by bleomycin stimulation, and Lacidipine treatment markedly restored the reduction of CXCR7 expression (Figures 3B,C). In addition, the levels of P38, p-P38, and inflammatory factors (VCAM1, Endoglin, MCP-1, and IL-6) were increased in bleomycin-stimulated ECs that were significantly inhibited by Lacidipine (Figures 3C–E).

### Lacidipine Protects ECs Against Cell Senescence Through the CXCR7 Pathway

**Figure 4:**
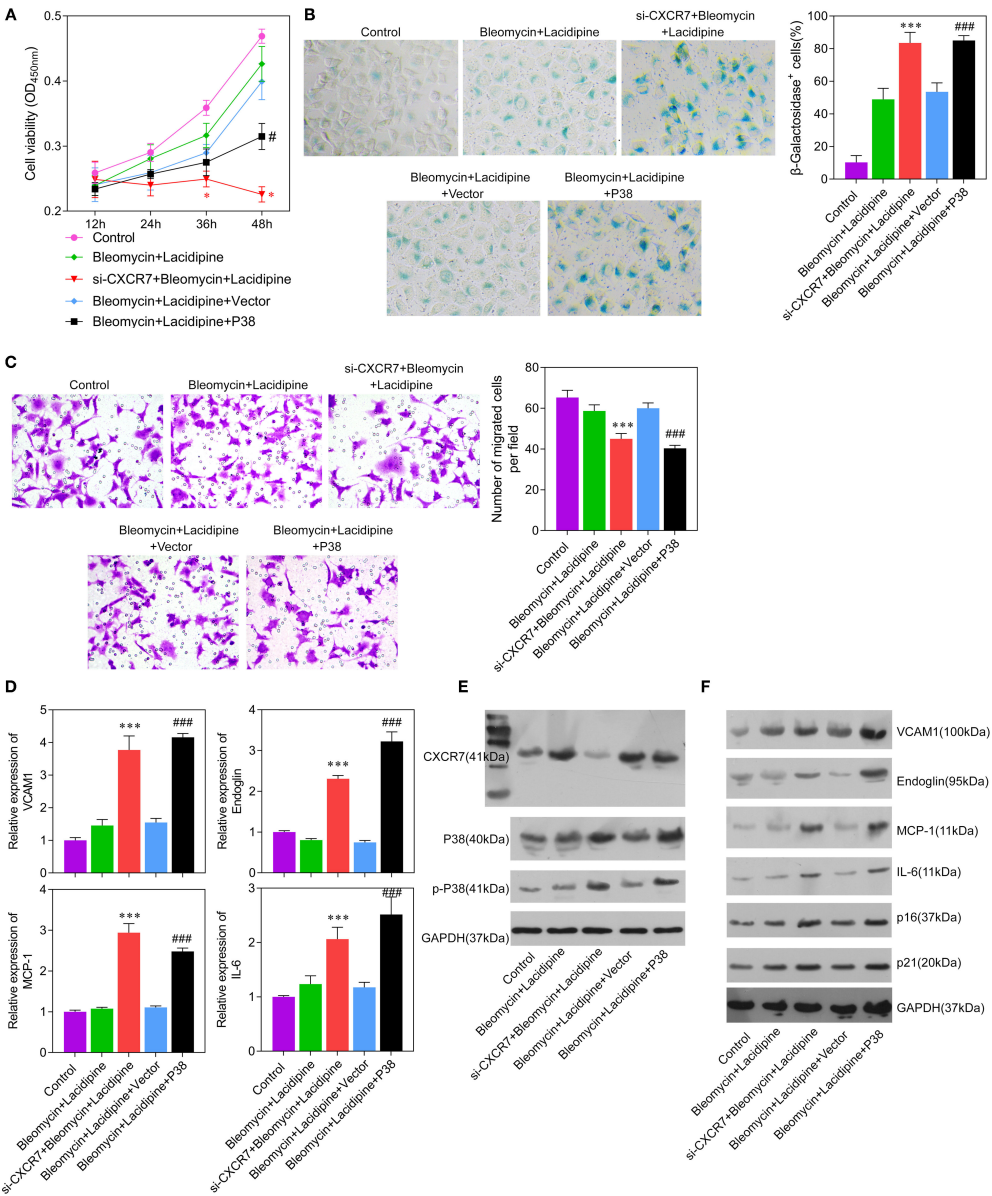
CXCR7 knockdown reversed the effects of Lacidipine on the *in vitro* function of ECs under senescence. ECs from the bleomycin + Lacidipine group were transfected with si-CXCR7 or overexpressed P38, respectively. Cell viability was determined using CCK-8 assay **(A)**. Cell senescence was determined through β-galactosidase staining **(B)**. Transwell assay was applied to analyze cell migration ability **(C)**. qPCR was performed to analyze the mRNA levels of VCAM1, Endoglin, MCP-1, and IL-6 **(D)**. Western blot was performed to analyze the protein levels of CXCR7, P38, p-P38, VCAM1, Endoglin, MCP-1, IL-6, P16, and P21 **(E,F)**. (Data are mean ± SD of three independent experiments. *p < 0.05, ***p < 0.001, compared with the bleomycin + Lacidipine group; #*p* < 0.05, ###*p* < 0.001, compared with the bleomycin + Lacidipine + Vector group).

To investigate whether the CXCR7 signaling pathway was the key regulator of Lacidipine-related endothelial protection, we specifically knocked down CXCR7 or overexpressed P38 in ECs *in vitro*, respectively. Our results showed that the cell viability (Figure 4A) and migration capacity (Figure 4C) were significantly impaired in the si-CXCR7 + bleomycin + Lacidipine and bleomycin + Lacidipine + P38 groups. The cells in senescence (Figures 4B,F) in the si-CXCR7 + bleomycin + Lacidipine and bleomycin + Lacidipine + P38 groups were remarkably increased compared with those in the bleomycin + Lacidipine group. Moreover, CXCR7 knockdown or P38 overexpression significantly increased the level of P38 and P38 phosphorylation and promoted the expression of VCAM1, Endoglin, MCP-1, and IL-6 (Figures 4D–F). These results indicated that CXCR7/P38 plays an important role in Lacidipine-related endothelial protection.

### CXCR7/P38/C/EBP-β Mediates the Anti-inflammation Effects of Lacidipine on ECs

**Figure 5:**
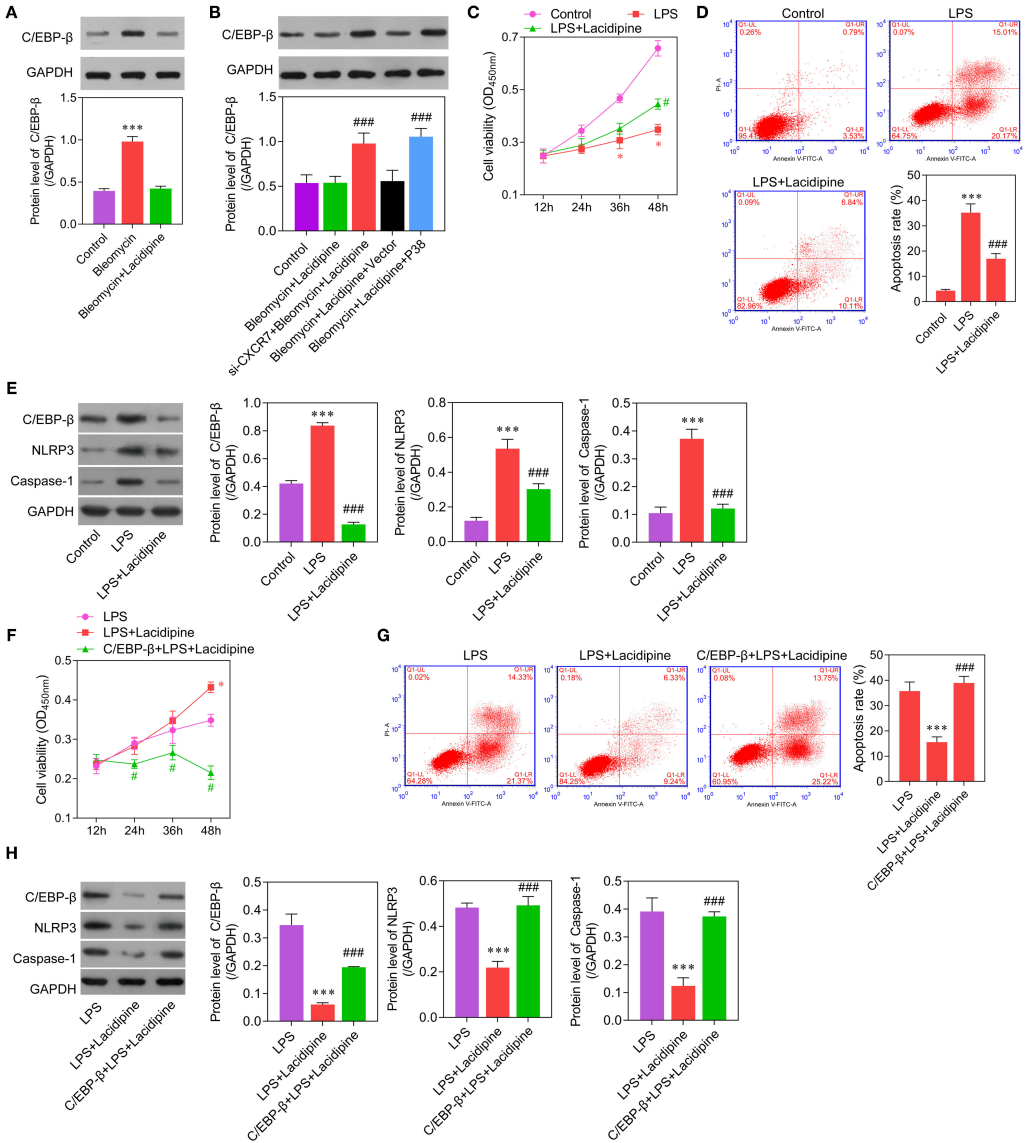
C/EBP-β was involved in Lacidipine-mediated anti-inflammation and anti-apoptosis effects on ECs. Cultured ECs were treated with Lacidipine, followed by bleomycin stimulation. Western blot was performed to analyze the level of C/EBP-β **(A,B)**. (***p < 0.001, compared with the bleomycin + Lacidipine group; ###*p* < 0.001, compared with the bleomycin + Lacidipine + Vector group). To further study the inflammatory activation, ECs were treated with Lacidipine, followed by LPS stimulation. Cell viability was determined using CCK-8 assay in ECs from the control, LPS, and LPS + Lacidipine groups **(C)**. Apoptotic cells were determined by flow cytometry in ECs from the control, LPS, and LPS + Lacidipine groups **(D)**. The protein expression of C/EBP-β, NLRP3, and caspase-1 was detected by Western blot in ECs from the control, LPS, and LPS + Lacidipine groups **(E)**. (Data are mean ± SD of three independent experiments. *p < 0.05, ***p < 0.001, compared with the control group; #*p* < 0.05, ###*p* < 0.001, compared with the LPS group). Cell viability was determined using CCK-8 assay in ECs from the LPS, LPS + Lacidipine, and C/EBP-β + LPS + Lacidipine groups **(F)**. Apoptotic cells were determined by flow cytometry in ECs from the LPS, LPS + Lacidipine, and C/EBP-β + LPS + Lacidipine groups **(G)**. The protein expression of C/EBP-β, NLRP3, and caspase-1 was detected by Western blot in ECs from the LPS, LPS + Lacidipine, and C/EBP-β + LPS + Lacidipine groups **(H)**. (Data are mean ± SD of three independent experiments. *p < 0.05, ***p < 0.001, compared with the LPS group; #*p* < 0.05, ###*p* < 0.001, compared with the LPS + Lacidipine group).

Considering the important role of C/EBP-β in inflammation, we further examined whether C/EBP-β participated in the CXCR7/P38-mediated anti-inflammatory effects of Lacidipine on ECs. Our results showed that the expression level of C/EBP-β was increased in ECs treated with bleomycin. Lacidipine treatment partly inhibited the increase of C/EBP-β induced by bleomycin in ECs, and CXCR7 knockdown or P38 overexpression in ECs abolished the effect of Lacidipine on C/EBP-β (Figures 5A,B). Furthermore, ECs were stimulated with Lipopolysaccharides (LPS) after Lacidipine treatment. As shown in Figure 5C, Lacidipine treatment notably improved the impaired cell viability induced by LPS in ECs. LPS-induced cell apoptosis in ECs was significantly reduced by Lacidipine treatment (Figure 5D). Western blot analysis further manifested that the upregulation of C/EBP-β, NLRP3, and caspase-1 induced by LPS was all markedly reduced by Lacidipine treatment (Figure 5E). We also performed gain-of-function assays in ECs from the LPS + Lacidipine group by C/EBP-β overexpression. As expected, C/EBP-β overexpression abrogated the protective effects of Lacidipine on cell viability (Figure 5F) and apoptosis (Figure 5G) in ECs stimulated with LPS. In addition, C/EBP-β overexpression led to the upregulation of NLRP3 and caspase-1 in the LPS + Lacidipine group (Figure 5H). These results demonstrated that Lacidipine suppressed the inflammatory activation and apoptosis in ECs through CXCR7/P38/C/EBP-β.

## Discussion

In this study, we demonstrated that Lacidipine protects ECs against oxidative stress, inflammatory activation, and cell senescence through the CXCR7/P38/C/EBP-β signaling pathway. Arterial hypertension is associated with increased levels of ROS that are important contributors to hypertension-related endothelial dysfunction and senescence (18–21). ROS explosion caused by the disorder of mitochondrial metabolism has been considered as the main cause of cell senescence (22). The increase in ROS leads to the increase in intracellular (DNA) damage and ultimately can result in the onset of apoptosis or the induction of cellular senescence in ECs (23). In our study, we found that Lacidipine effectively suppressed the ROS production, NADPH activity, and expression of NOX1/NOX2 in ECs. Under H_2_O_2_-induced oxidative stress, we observed decreased cell viability and migration capacities in ECs that can be restored by Lacidipine treatment. Studies from other groups also reported that Lacidipine displays antioxidant activity, which is effective for preventing endothelial dysfunction (13, 15). By using bleomycin to induce EC senescence *in vitro*, we found that Lacidipine-pretreated ECs were also partially resistant to cell senescence. Taken together, our study demonstrated that Lacidipine exerts additional beneficial effects on hypertension-related endothelial injury by attenuating EC oxidative damage and cell senescence.

To further decipher the molecular mechanisms underlying Lacidipine-related protective effects, we specifically knocked down the expression of CXCR7 in ECs using si-RNA transfection. We observed that CXCR7 deficiency abolished the protective effects of Lacidipine on the function of ECs *in vitro* under the stimulation of bleomycin. As we know, CXCR7 is essential for the regulation of cellular biological functions (24). In some cancer cells, CXCR7 was involved in the resistance to apoptosis (25, 26). Yu et al. found that CXCR7 enhances ovarian cancer cell invasion through the P38 MAPK pathway (27). More importantly, CXCR7 plays a critical role in the regulation of EPC growth and survival (28). In the present study, we demonstrated that CXCR7 attenuated the oxidative injury and cell senescence in ECs through the inhibition of P38 phosphorylation. Moreover, the overexpression of P38 can inhibit Lacidipine-related beneficial effects on ECs. These findings suggest that the CXCR7/P38 axis is essential for Lacidipine-related EC protection.

Given the important role of P38 in the regulation of inflammation (29), we further explored the effect of the CXCR7/P38/C/EBP-β pathway on the inflammatory activation in ECs. We found that the inhibition of CXCR7 or overexpression of P38 led to the increase of C/EBP-β level. In addition, Lacidipine could effectively suppress LPS-induced inflammation and apoptosis by downregulating C/EBP-β expression in ECs. Studies showed that C/EBP-β is crucial for the inflammation and apoptosis associated with the pathogenesis of aging (11, 12). According to the report by Jain et al. (30), the C/EBP family of transcription factors may be involved in essential hypertension. Considering the association between inflammation and EC senescence, our results indicate that the CXCR7/P38/C/EBP-β pathway was involved in Lacidipine-related protection against endothelial inflammatory injury and apoptosis.

In summary, to the best of our knowledge, the present study demonstrates for the first time that Lacidipine attenuated endothelial oxidative injury, inflammatory activation, and senescence associated with hypertension by regulating the CXCR7/P38/C/EBP-β pathway. Our findings provide a new insight for the molecular mechanisms underlying Lacidipine-mediated EC protection that might be helpful for developing novel therapeutic strategies for hypertension-related vascular disease.

## Data Availability Statement

The original contributions presented in the study are included in the article/supplementary materials, further inquiries can be directed to the corresponding author/s.

## Ethics Statement

The animal study was reviewed and approved by the First Affiliated Hospital of Sun Yat-sen University.

## Author Contributions

XL, ZH, ZW, and SX contributed to the design, data collecting, and manuscripts preparation of this study. YZ and XS contributed to the *in vitro* and molecular study. FL contributed to the animal experiment. All authors contributed to the article and approved the submitted version.

## Conflict of Interest

The authors declare that the research was conducted in the absence of any commercial or financial relationships that could be construed as a potential conflict of interest.
